# Formulation and biochemical evaluation of designer diet enriched with botanicals for bone health

**DOI:** 10.1002/fsn3.1680

**Published:** 2020-05-29

**Authors:** Ammara Yasmeen, Muhammad Sajid Arshad, Rabia Shabir Ahmad, Farhan Saeed, Ali Imran, Faqir Muhammad Anjum, Hafiz Ansar Rasul Suleria

**Affiliations:** ^1^ Institute of Home and Food Sciences, Faculty of Life Sciences Government College University Faisalabad Pakistan; ^2^ Pakistan Council of Scientific and Industrial Research Lahore Laboratories Complex Lahore Pakistan; ^3^ Vice Chancellor University of The Gambia (UTG) Banjul Gambia; ^4^ Department of Agriculture and Food Systems The University of Melbourne Melbourne Vic. Australia

**Keywords:** amino acid profile, antioxidant potential, bone strength, botanical‐based nutrients, fatty acid profile

## Abstract

The study designed to evaluate the role of sesame, fenugreek flax, and nuts seeds as best alternatives for therapeutic agents to improve bone mineral status. Effect of these plant seeds on proximate composition, antioxidant potential, mineral, fatty acid, and amino acid profile of supplements was studied. The sesame, fenugreek, and flaxseeds were incorporated with nuts at level of 35% to develop supplement. Improved contents of protein, fat, and NFE were recorded in range of 25.72–32.23, 18.92–23.15, and 34.25%–39.59%, correspondingly. The antioxidant potential of supplements is high (450.56 mg GAE/ 100g) prepared with fenugreek followed by flaxseeds and sesame seeds. Calcium as fundamental bone mineral found greater (525.15%–790.21%) in seed‐based supplement. Enhanced contents of Oleic, linoleic, and linolenic acids ranged 17.32–41.78, 21.72–44.23, and 1.00%–47.12%, respectively. Nutritional enriched supplements may be best alternatives for improving bone health by lowering osteoclast and increasing osteoblast mechanism in bone development.

## Graphical abstract

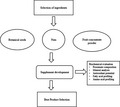

## 1 INTRODUCTION

Our skeleton is a major module of body as it provides a strong defensive fundamental structure. Due to which body is able to cope with daily routine and maintain whole physique. Regeneration and maintenance of bone tissues is a continuous progression. Bone is not merely a mineral reservoir but much of its composition is protein, fatty acid, and polysaccharides which is an organic segment. And it also comprehended a complex of nerves, blood vessels, and marrow at center. Bones are major reservoir of calcium about 99 percent of whole body (Song, 2017), which is available in form of hydroxyapatite. Other minerals (including Ca, P, Mg, Vitamin K, and Vitamin D) constitute the inorganic part of bone for its development and maintenance.

One of several risk factors for reducing bone strength and several disorder is low bone frame, and these factors may characterized as skeletal and nonskeletal. Most cases of low bone density occur in aged subjects, particularly postmenopausal women indeed, a decline of estrogen levels in females at menopause leads to loss of bone mass and strength and contributes to the development of bone diseases such as osteopenia and osteoporosis. Skeletal factors involve the alteration in biomechanical strength determining the bone size and shape act without interference of any other related issue (Weaver et al., 2016). Environmental factors, especially dietetic behavior and routine diet plan are major contributing aspect of bone mass loss. Alcohol and nicotine taking, low physical activity, dependency on medication are determinants of these factors (Martha et al., 2019). Diet is one of several important parameters for optimum bone density and treating of bone ailments. As the significance of calcium and vitamin D is concerned, it has been much discussed in previous studies in regards to improve bone health (Peters & Martini, 2010). Balanced nutrition enriched diet besides fulfilling daily energy requirement also play an important role in providing essential micronutrients, which ultimately helps to maintain bone mass and bone mineral density (Anastasilakis *et al*., 2017).

Nutritional therapy has abundant impact on bone health and key role in management of bone disorders like osteopenia, osteoporosis, and arthritis. It is an amendable element to strengthen bone health and inhibit the initiation of bone‐related issue. Furthermore, studies have shown that diets high in fruits and vegetables have positive effects on bone mineral status and that nutrients and vitamins, including vitamin K, vitamin C, phosphorus, potassium, magnesium, protein, and sodium, are important for the maintenance of optimal bone health (Prynne et al., 2006). The findings of many studies have related the intake and/or serum levels of several vitamins, including A, B complex, C, E, and K, as well as the homocysteine (Hcy) level, to bone health in animals and in humans.

Plant‐based curative composites have positive effects against various human diseases without any hazardous side effects. These compounds are widely used to cure the disorders along providing essential nutrients and improving performance (Shirwaikar, Khan, Kamariya, Patel, & Gajera, 2010). Plant‐based compounds are being used in many traditional systems mostly in rural areas but on increasing urbanization synthetic foods and medicines has been in practice. Instead, plant‐derived medication has been investigated much safer than synthetic pharmacological elements. Out of many plant‐based sources sesame, fenugreek and flaxseeds are important and generally ignored food stuffs which can contribute to strengthen the body potential against disorders.

Sesame seed is delicious flavored food containing health benefitting nutrients like essential fats, minerals, antioxidants, and vitamins. Sesame seed is considered to be helpful for its potential to provide recommended daily allowance (RDA) of certain nutrients like calcium, phosphorus, iron, magnesium, and potassium. As a study revealed that sesame seed provides 100% of the recommended daily allowance (RDA) for manganese and potassium, 57%–65% of the RDA of phosphorus and iron, and 13%– 35% for zinc, calcium, and copper while its recommended daily intake is 25 to 50 grams (Pathak, Rahman, Bhagawati, & Gogoi, 2017). Lignan glycosides have naturally potential against oxidation as antioxidant which is rich in sesame seed. This antioxidant ability helps to prevent bone inflammation consequently prevent bone resorption and increase bone formation (Kim, Lee, & Lee, 2006).

Fenugreek (*Trigonella foenum‐graecum Linn*) is seasoning ingredient used in foods have much medicinal and therapeutic value. Fenugreek seed is complex of many dietary nutritional components like amino acids, fatty acids, vitamins, and saponins such as disogenin, flavonoids, polysaccharides, and glycosides recommended for therapeutic use (Umesh, Najma, & Baquer, 2014). Fenugreek seed extract have positive effect on lowering the consumption of dietary fat in humans, and it highly modifies the feeding behavior which is responsible for weight management. This investigation is a major factor for lowering bone loss by weight controlling especially in females. Fenugreek has antiosteoporotic ability in presence disogenin and helps to reduce process of osteoclast which contribute to bone mass loss ultimately (Nesma, El‐ Nasr, El‐Denshary, Mahmoud, & Nofal, 2018).

Flaxseed is a oil containing kernel rich in essential fatty acids, dietary fiber, and proteins. As containing high amounts of omega 3 fatty acids and α‐linolenic acid, it contributes to stimulation of osteoblast process during which bone mass substance secreted during estrogen deficiency. Linolenic acid is a precursor of eicosapentaenoic acid is higher in flaxseed enriched diet (56%) than fish is effective in reducing bone loss consequently preventing osteoporosis (Boulbaroud, Mesfioui, Arfaoui, Quichou, & El Hessni, 2008). Phosphorus and magnesium are major mineral elements for bone strength, and composition is in higher amounts in flaxseed (Branski, Fasano, & Troncone, 2006).

All available treatments for bone‐related diseases include pharmaceutical pharmacological and synthetic agents which act by reducing rate of bone mineral loss and increasing bone potential to avoid fracture risk. But these therapeutic managements have side effects like gastrointestinal infection, hypercalcemia, dyspepsia. Thus, there is a need to explore natural plant‐based sources for enhancing skeleton strengthening before commencing bone losses.

## 2 MATERIAL AND METHODS

### 2.1 Procurement of raw materials

Fenugreek, Sesame, and Flaxseeds of autumn season were procured from Ayub Agricultural Research Institute (AARI), Faisalabad, Pakistan. Other ingredients were purchased from local market. Analytical reagents and HPLC grade standards were obtained from Merck (Darmstadt, Germany) and Sigma‐Aldrich.

### 2.2 Preparation of raw materials

The seeds of sesame, fenugreek, and flaxseed were cleaned by sieving followed by sorting. Afterward, milling of seeds along with other ingredients was carried out using Laboratory (Perten‐3100) mill to get straight grade fine powdered sample. The resultant powder was stored in air tight containers prior to analysis.

### 2.3 Product development

Three different supplement formulations were developed from the selected raw materials. These supplements comprise similar composition except one major ingredient. As first, second, and third supplement contains a major ingredient like sesame seed, fenugreek, and flaxseed respectively as shown in Table 1. All ingredients were mixed homogenously. Grapes, papaya, and mud apple concentrate were prepared after peeling, slicing, and grinding one by one. The concentrate was then freeze dried to get powder to use it as flavor. Finally this powder was mixed in their respective formulation.

**Table 1:** Composition of nutritional supplement for bone health

Ingredients (percent by weight)	Treatments		
	T1 (SS)	T2 (FS)	T3 (FXS)
Major ingredient	35	35	35
Almond	10%	10%	10%
Cashew nut	10%	10%	10%
Walnut	10%	10%	10%
Dextrose	25%	25%	25%
Dates powder	5%	5%	5%

Note

T1 contain Sesame seed and also 5% grapes concentrate powder; T2 contain Fenugreek seed and also 5% papaya concentrate powder; T3 contain Flaxseed and also 5% mud apple concentrate powder.

### 2.4 Characterization of supplement formulations

#### 2.4.1 Proximate composition

Supplement samples were analyzed for moisture, crude protein, crude fat, crude fiber, ash, and nitrogen‐free extract by using triplicate samples (AOAC, 2016).

#### 2.4.2 Mineral profile

The samples were subject to mineral assay through wet digestion considering the protocols of AOAC (2016). For the estimation of sodium and potassium, Flame Photometer‐410 will be used while calcium, iron, magnesium, Phosphorus measured through Atomic Absorption Spectrophotometer.

#### 2.4.3 Fatty acid profile

Fatty acid methyl esters (FAMEs) were prepared from the oil samples according to protocol described by (Lutterodt, Slavin, Whent, Turner, & Yu, 2011). Briefly, 1 mg of oil was reacted with 0.1 mol/L NaOH‐MeOH for 5 min, followed by reacting with 1.1 mol/L HCl‐MeOH for 5 min at ambient temperature. After adding water to stop the reaction, FAMEs were extracted with iso octane. GC analysis was conducted with a Shimadzu GC‐2010 equipped with a FID and a Shimadzu AOC‐20i auto sampler.

#### 2.4.4 Amino acid analysis

Amino acid analysis of research samples was valued following the method described by (Adebisi, Tephan, & Chinedu, 2017). Analysis of defatted samples carried out with the aid of HPLC equipped with UV 338 nm detector, Column C18, 2.5 × 200 mm, 5 µm column and mobile phase of 1:2:2 (100 mM Sod sulfate, pH 7.2; Acetonitrile; Methanol (v/v/v) at a flow rate of 0.45ml/minute and an operating temperature of 40°C.

#### 2.4.5 Free radical scavenging ability

Free radical scavenging activity of supplement formulations was estimated using 1, 1‐ diphenyl‐2‐picrylhydrazyl (DPPH) (Cat# D9132) as described by Muller, Frohlich, and Bohm (2011). The inhibition of free radicals by DPPH was calculated through following expression:

#### 2.4.6 Total phenolic contents

Total phenolic content determination was performed for prepared supplement by using procedures described by Singleton, Orthofer, and Lamuela‐Raventos (1999). Decant two mL of aqueous extract in a 10 ml volumetric flask, then added 5 ml of distilled water followed by 0.3 ml of 5% NaNO_2_ and after 5 min – 0.6 ml of 10% AlCl_3_ was added. After another 5 min stay, 2 ml of 1 M NaOH was added and volume was made up to 10 ml with distilled water. Take one mL from this solution after mixing this solution, add 9 ml distilled water and measured absorbance by using spectrophotometer at 765 nm. Total phenolic content was calculated and expressed as Gallic Acid Equivalent (mg Gallic Acid/100g).

#### 2.4.7 Total favonoids

Total flavonoids contents of supplements were evaluated using the procedure described by Zhishen, Mengcheng, and Jianming (1999). Took 2ml of aqueous extract of sample in a 10 ml volumetric flask, and added 0.3 ml of 5% NaNO_2_ then 5 ml distilled water and after 5 min – 0.6 ml of 10% AlCl3 was added. A 2 ml of 1 M NaOH was added after 5 min stay, and volume was made up to 10 ml with distilled water. Take one mL from this solution with 9 ml distilled water and measured absorbance by using spectrophotometer at 510 nm. Total flavonoids were expressed as Catechin equivalents per dry matter.

#### 2.4.8 Ferric reducing antioxidant power (FRAP)

Ferric reducing antioxidant power was measured by using the method of Muller et al. (2011). An aliquot of prepared supplement samples extract (50 μl each) was taken with 3 ml of FRAP reagent following incubation at 37°C for 30 min. The increase in absorbance was noted at 593 nm using Spectrophotometer. The results were compared with the calibration curve, prepared by using various concentrations of trolox as standard.

### 2.5 Statistical analysis

The results for chemical assays were expressed as mean ± *SD* of three independent analyses. One‐way analysis of variance (ANOVA) was used to verify the difference between the sample groups. Statistical significance of the difference was tested while using LSD test at *p* ≤ .05. The data obtained from all the determinations were statistically analyzed by using the statistical software Statistix 8.1 (Informer Technologies).

## 3 RESULTS AND DISCUSSIONS

### 3.1 Proximate composition

As balanced nutrition diet is essential for better human health. A food compound of various ingredients delivers minerals, vitamins, and macro nutrients required for body well‐being, which play crucial role against diseases caused by malnourishment consequently reducing risk of numerous ailments. The results depicted that moisture, ash, fat, protein, fiber, and NFE contents significantly different with respect to different treatments and is given in Table 2, which showed the maximum protein contents in flaxseed‐based supplement i‐e 32.23 following by fenugreek and sesame seed‐based supplement 28.56 and 25.72, respectively. Flaxseed fortified product showed increased amount of protein as studied by Patil, Sheety, Todkar, and Bodhankar (2013); Masoodi, Aeri, and Bashir (2012). Protein is most important nutrient to enhance body immunity and resistance against initiation of diseases.

**Table 2:** Proximate composition of prepared supplement depending on the relevant references

Parameters	Supplement %		
	T1 (SS)	T2 (FS)	T3 (FXS)
Moisture	5.93 ± 0.03^b^	6.00 ± 0.02^a^	5.78 ± 0.03^c^
Ash	2.15 ± 0.11^a^	2.05 ± 0.08^b^	1.82 ± 0.09^c^
Fat	23.15 ± 0.21^a^	18.92 ± 0.19^b^	22.57 ± 0.25^a^
Protein	25.72 ± 0.20^c^	28.56 ± 0.19^b^	32.23 ± 0.21^a^
Fiber	3.46 ± 0.30^a^	3.18 ± 0.15^c^	3.35 ± 0.22^b^
NFE	39.59 ± 0.19^b^	41.29 ± 0.15^a^	34.25 ± 0.20^c^

Note

All data are the mean ± *SD* of three replicates. Means followed by different letters (a, b, and c) within the same row are significantly different (*p* < .05) from each other using a one‐way analysis of variance (ANOVA) and LSD test.

Abbreviation: NFE, Nitrogen‐Free Extract.

High protein diets are key to increase the anabolic insulin like growth factors which is resultantly play a crucial role in bone structure development. These sufficient proportioned protein diets are accompanying with proper calcium absorption. On contrary, lower protein content diet consumption reduce the calcium absorption results rise in parathyroid hormone rise, which aid the bone mass loss (Heaney & Layman, 2008). On the basis of its nutrition, botanical constituted diet have ability to change acid–base status making alkaline which is favorable for bone health (James et al., 2016). Essential fatty acid containing fat contents of current study ranged between at maximum of 23.15% in sesame seed‐based supplement and at minimum of 18.92% in fenugreek‐based supplement. Similar results found by (Kasaye & Kumar, 2015) in which the crude fat content of samples prepared from 15% fenugreek ranged from 13.64% to 17.02%. A study revealed that diet with ample amount inclusion of walnuts, walnut flaxseed versus an average American diet decreased a marker of bone resorption (Orchard, Pan, Cheek, Ing, & Jackson, 2012).

NFE contents in any of food formulation or supplement are responsible for improved flavor and its texture. It also plays a vital role in storage stability of the product particularly shelf life by maintaining total solids. NFE contents of sesame seed‐based supplement are observed to be 39.59 ± 0.19% which is little lower and comparable to 45.56 ± 0.19 calculated for 25% sesame flour supplemented bar 45.56 ± 0.19 (Abbas, Sharif, & Ejaz, 2016). Fenugreek seed‐based supplement showed higher NFE contents of 41.29%±0.15 in the presence of nuts in similarity of 35.14–41.95 observed by (Man et al., 2019) of bread supplemented with fenugreek seed flour.

### 3.2 Mineral profile

Minerals are important constituent of human bone composition. Any reduction of particular mineral quantity in human serum affects the bone strength quality. Absorption of these minerals depends upon the whole composition of diet taken. The results showed that mineral profile of prepared supplements has a significant difference among each other and is given in Table 3. In present exploration, sesame seed‐based supplement has mineral values 790.21, 629.00, 351.23, 9.87, 473.5, and 39.42 of calcium, phosphorus, magnesium, iron, potassium, and sodium, respectively (Table 2). These findings are similar to that of Abbas et al., 2016. Likewise calcium, phosphorus, magnesium, iron, potassium, and sodium content values of fenugreek containing supplement are 425.15, 453.23, 373.47, 13.55, 321.37, and 37.25, respectively. But mineral profile is little lower than that of flaxseed‐based supplement except potassium 616.7 and magnesium 394.5 contents which are observed to be higher than sesame seed‐ and fenugreek seed‐based product. As best calcium–phosphorus ratio is for bone structure considered 2:1 (Nemati, Huda, & Ariffin, 2017) which has been recorded for sesame seed‐based supplement.

**Table 3:** Mineral profile of prepared supplement

Minerals	Supplement mg/100g		
	T1 (SS)	T2 (FS)	T3 (FXS)
Calcium	790.21 ± 0.25^a^	525.15 ± 0.30^c^	573.17 ± 0.33^b^
Phosphorus	425.00 ± 0.30^b^	453.23 ± 0.27^a^	311.20 ± 0.40^c^
Magnesium	151.23 ± 0.18^c^	373.47 ± 0.22^b^	394.50 ± 0.15^a^
Iron	9.87 ± 0.33^c^	13.55 ± 0.37^a^	12.73 ± 0.25^b^
Potassium	173.50 ± 0.19^c^	321.37 ± 0.30^b^	616.70 ± 0.21^a^
Sodium	39.42 ± 0.21^a^	37.25 ± 0.17^b^	29.37 ± 0.30^c^

Note

All data are the mean ± *SD* of three replicates. Means followed by different letters (a, b, and c) within the same row are significantly different (*p* < .05) from each other using a one‐way analysis of variance (ANOVA) and LSD test.

Potassium is one of the important minerals in any health improvement supplement playing crucial role in cell membrane potential and ion transport through membrane. Potassium and magnesium of flaxseed powder product were reported 200.97 and 125.86 respectively by Kaur and Das (2015), which is relatively is lower than that of present study due to the addition of comparable higher amount of flaxseed powder along in presence of nuts. Calcium, potassium, and magnesium of walnut in a study reported as 1,062, 2,771, and 1,426 respectively on whole weight basis (Cindric, Zeiner, & Hlebec, 2018) which is major mineral enrichment ingredient in present study as a minor amount ingredient.

Fenugreek having high nutritious value can be used as value addition ingredient in many products like bakery, snacks and other cereal‐based fortified flours. To obtain multiple benefits, it is a mineral enriched daily diet constituent which will help in body metabolism. Bread and biscuits prepared by adding various percentages of fenugreek powder showed a range of minerals like calcium, magnesium, iron, and zinc from 5.71 to 78.04 (Kasaye & Kumar, 2015) But in present study 35% fenugreek seed‐based supplement with inclusion of nuts the range of mineral profile is much greater like 616.7% potassium to least 12.73% iron.

### 3.3 Amino acid composition

The amino acid composition has a significant difference with respect to treatments and is given in Table 4. Glutamic acid ranked the highest concentration in sesame (19.23g/100g), fenugreek seed (17.86g/100g), and flaxseed (16.87g/100g)‐based supplement. This observation is similar to that of reflected by (Fasuan, Saka, Gbadamosi, & Omobuwajo, 2018). Amino acid profile results showed least contents amino acids are methionine, cysteine, and tryptophan. Among the essential amino acids, lysine is estimated to be highest in all three supplements 8.05 g/100g in T1, 8.90g/100g I T2, and 6.12g/100g in T3 than other. These findings are opposite to observed by Fleddermann et al. (2013) who concluded the higher essential amino acid is leucine. Valine, tryptophan, and isoleucine contents of fenugreek are similar to those evaluated by Hegazy (2011) in burgers using various concentrations of fenugreek.

**Table 4:** Fatty acid profile of prepared supplements (%)

Fatty acids	T1 (SS)	T2 (FS)	T3 (FXS)
16:0 (Palmitic)	9.68 ± 0.21^b^	11.55 ± 0.13^a^	7.53 ± 0.25^c^
16:1 (Palmitoleic)	0.25 ± 0.01^b^	0.33 ± 0.01^a^	0.23 ± 0.01^b^
18:0 (Stearic)	4.37 ± 0.12^b^	3.17 ± 0.07^c^	5.45 ± 0.16^a^
18:1 (Oleic)	41.78 ± 0.17^a^	19.28 ± 0.23^b^	17.32 ± 0.31^b^
18:2 (Linoleic)	42.32 ± 0.26^a^	44.23 ± 0.27^a^	21.72 ± 0.14^b^
18:3 (Linolenic)	1.00 ± 0.02^c^	20.62 ± 0.11^b^	47.12 ± 0.34^a^

Note

All data are the mean ± *SD* of three replicates. Means followed by different letters (a, b, and c) within the same row are significantly different (*p* < .05) from each other using a one‐way analysis of variance (ANOVA) and LSD test. 16:0, palmitic acid; 16:1, palmitoleic acid; 18:0, stearic acid; 18:1, oleic acid; 18:2, linoleic acid; 18:3, linolenic Acid.

Essential amino acids have positive effects on bone status which cannot be synthesized by human body. Deficiency of these amino acids can improved by micronutrient supplemented diets. It has been observed by recent study that micronutrient supplementation positively affects collagen building during fracture healing. A study revealed that patients of bone disorder cured with micronutrient supplemented diet plan containing vitamin C, lysine, proline, and vitamin B6 helped the quick recovery during fracture healing (Karpouzos, Diamantis, Farmaki, Savvanis, & Troupis, 2017). Amino acid has impact on osteoblast and osteoclast differentiation positively. In a study, arginine proved constructive effect on both osteoblastic activity and osteogenesis by increasing IGF‐1 production may improve bone formation mechanism (Robert, Hamrick, & Isales, 2016).

### 3.4 Fatty acid profile

Fatty acids have important role in bone metabolism and potential to resist against bone loss preventing osteoporosis. The fatty acid profile has significant difference with respect to treatments and is given in Table 5. Supplement prepared in current study showed a positive trend of fatty acids profile for bone health improvement. Omega 3 fatty acids are one of the classes of fatty acids having potential of improving bone turnover. Plants and nuts are major sources of omega 3 fatty acids (Ricard, Mònica, & Salas‐Salvadó, 2016). Fatty acid composition of sesame seed‐based supplement exhibited best of linoleic 42.32, Oleic 41.78, and palmitic acid 9.68 values, which are slightly higher than estimated by Elleuch *et al*. (2007). Reported values of sesame seed along with nuts supplement are also comparable with sesame seeds fatty acid composition evaluated by Biglar et al. (2012).

**Table 5:** Amino acid profile of prepared supplements (g/100g)

Amino acid	T1 (SS)	T2 (FS)	T3 (FXS)
Aspartic acid	5.10 ± 0.12^b^	5.45 ± 0.11^b^	8.72 ± 0.15^a^
Glutamic acid	19.23 ± 0.24^a^	17.86 ± 0.32^b^	16.87 ± 0.27^b^
Cystine	1.10 ± 0.04^c^	1.53 ± 0.11^a^	1.16 ± 0.10^b^
Serine	6.15 ± 0.13^a^	5.93 ± 0.32^b^	5.12 ± 0.16^c^
Tyrosine	4.75 ± 0.14^a^	2.10 ± 0.11^c^	3.10 ± 0.14^b^
Phenyalanine*	5.37 ± 0.16^a^	4.68 ± 0.15^c^	4.90 ± 0.12^b^
Isoleucine*	4.66 ± 0.11^a^	4.17 ± 0.14^b^	4.05 ± 0.19^c^
Leucine*	7.46 ± 0.24^a^	7.30 ± 0.16^b^	6.27 ± 0.11^c^
Histidine*	2.84 ± 0.16^a^	2.75 ± 0.12^b^	2.68 ± 0.12^c^
Valine*	4.44 ± 0.09^c^	5.12 ± 0.16^a^	4.70 ± 0.16^b^
Tryptophan*	1.20 ± 0.05^b^	1.17 ± 0.05^b^	1.90 ± 0.06^a^
Methionine*	1.93 ± 0.07^b^	2.16 ± 0.13^a^	1.86 ± 0.17^c^
Proline	9.67 ± 0.21^a^	8.55 ± 0.15^b^	6.50 ± 0.24^c^
Glycine	3.43 ± 0.12^c^	3.70 ± 0.13^b^	5.87 ± 0.24^a^
Alanine	3.83 ± 0.14^c^	4.00 ± 0.14^b^	5.10 ± 0.12^a^
Arginine	4.22 ± 0.26^b^	3.77 ± 0.12^c^	8.72 ± 0.36^a^
Lysine*	8.05 ± 0.34^b^	8.90 ± 0.28^a^	6.12 ± 0.15^c^
Threonine*	6.55 ± 0.15^a^	5.85 ± 0.13^b^	5.00 ± 0.11^c^
Total Essential amino acids*	42.5 ± 0.28^a^	39.9 ± 0.41^b^	37.48 ± 0.32^c^
Total nonessential amino acids	57.48 ± 0.36^b^	52.89 ± 0.34^c^	61.16 ± 0.33^a^

Note

All data are the mean ± *SD* of three replicates. Means followed by different letters (a, b, and c) within the same row are significantly different (*p* < .05) from each other using a one‐way analysis of variance (ANOVA) and LSD test.

^*^ Total Essential amino acids.

As Table 3 presents the fatty acid composition obtained by gas chromatographic analysis of the methyl esters of the total lipids of prepared supplement. Most abundant fatty acid contents in flaxseed are oleic, linoleic, and linolenic acid 17.32, 21.72, and 47.12 respectively recorded by Boulbaroud et al. (2008) compared with existing study results calculated for supplemented flaxseed with other nutritional ingredients. Differences are due to nuts like cashew and walnuts containing essential fatty acids (Ryan, Galvin, Connor, Maguire, & Brien, 2006). Fenugreek supplement showed unsaturated fatty acid values for oleic, linoleic, and linolenic are 19.28, 44.23, and 20.62 respectively compared with report by Sulieman et al. (2008) with improvement in fatty acid profile in the presence of nuts and fruit sources.

### 3.5 Antioxidant potential

The results regarding the total phenolic content (TPC), free radical scavenging activity (DPPH), total flavonoids, and FRAP showed significant difference among different treatments and is given in Table 6. The results indicated the highest value 450.00 mg GAE/100g in T2 (fenugreek‐based supplement) followed by 156.00 mg GAE/100g in T3 and 123.23 mg GAE/100g in T1. The principal mechanism of antioxidant is scavenging free radicals by transferring hydrogen atom. Results of antioxidant analysis revealed that amount of total phenolic contents not proportional with DPPH activity of scavenging free radicals. This finding is similar to that of Kaur and Das (2015). Table 3 showed the higher value of DPPH activity in T2 (45.00%) and lowest in T3 (32.95). Values of fenugreek‐based supplement estimating higher gallic acid equivalent and its DPPH Activity much similar calculated in fenugreek containing bread by Afzal, Pasha, Zahoor, and Nawaz (2016).

**Table 6:** Total phenolics, Flavonoids, antioxidant capacity, and FRAP contents of prepared supplements

Treatment	TPC (mg GAE/ 100g)	DPPH (%)	FRAP (μmol trolox Eq/100g)	Total Flavonoids (mg CE/g)
T1 (SS)	120.23 ± 5.12^c^	38.56 ± 6.15^b^	2.45 ± 3.17^c^	1.85 ± 1.56^c^
T2 (FS)	450.56 ± 2.52^a^	45.00 ± 5.70^a^	5.71 ± 7.74^a^	3.75 ± 2.10^a^
T3 (FXS)	156.00 ± 6.35^b^	32.95 ± 4.13^c^	3.72 ± 6.16^b^	2.13 ± 1.35^b^

Note

All data are the mean ± *SD* of three replicates. Means followed by different letters (a, b, and c) within the same row are significantly different (*p* < .05) from each other using a one‐way analysis of variance (ANOVA) and LSD test.

Abbreviations: DPPH, 2,2‐diphenyl‐1‐picrylhydrazyl, free radical scavenging activity; FRAP, Ferric reducing antioxidant powder; GAE, Gallic acid equivalents; TPC, Total phenolic content.

Ferric reducing antioxidant power has been observed to be higher in fenugreek‐based supplement (T2) i‐e 5.71(μmol trolox Eq/100g) followed by flaxseed‐ (3.72) and sesame seed‐based supplement (2.45). In comparison with other antioxidant potential methods which determines the rate of inhibition of active agent, FRAP is the only assay that uses an antioxidant as reductant by redox reaction and estimate the antioxidants directly in sample (Apak, Ozyurek, Guçlu, & Çapanoglu, 2017). In a study reported by Kenny, Smyth, Hewage, and Brunton (2013), frap evaluation of fenugreek seeds powder distinctly showed value of 0.135 to 77.352 TE mg/g, which is comparable to current evaluation of fenugreek with various nuts. FRAP value of flaxseed powder extract estimated to be 0.58 to 1.08 mg TE/g by Deng et al. (2017), which is lower than that of supplement prepared in current study by the addition of nuts as 3.72.

Flavonoids are bioactive polyphenol and one of the important nutrient found abundantly in fruits, vegetables, nuts, and seeds. Flavonoids are class of antioxidant increases potential of the diet rich by these nutrients to minimize the oxidative stress. Flavonoids in tea have been reported to protect against hip fracture in late age years. Much of the studies on cellular model and animal trials has established the strong connotation between flavonoids and bone status (Hardcastle, Aucott, Reid, & Macdonald, 2011). Sesame seed being adequately nutritious has been esteemed as a food additive capable of having potential against many diseases. Table 5 presents the total antioxidant potential including Total flavonoids, which indicated the lower flavonoids value of 1.85 ± 1.56 in sesame seed‐based supplement than other treatments. This record is comparable and lower than 30% sesame‐supplemented diet calculated by William, Faulet, Koné, Gnanwa, and Kouamé (2018). Flaxseed‐based supplement (35%) contents calculated in current study are (2.13 ± 1.35 mg CE/g) in the presence of nuts and fruit ingredients, which is more than flaxseed reported flavonoid contents (35–70 mg/ 100 g) (Katare, Saxena, Agrawal, Prasad, & Bisen, 2012). Fenugreek seed is comparatively a good source of bioactive components like alkaloids, flavonoids, and saponins (Kasaye & Kumar, 2015) and attributed to antioxidant potential. Flavonoid contents of fenugreek‐supplemented formulation depict the higher value (3.75 ± 2.10) than other treatments.

## 4 CONCLUSION

This study focused on the formulation and biochemical evaluation of plant‐based nutrients against bone health. The outcomes of current study revealed that Sesame, fenugreek, and flaxseeds are good sources of proteins, fats, NFE. Increased protein contents in prepared supplements observed more in (T3) but differ in mineral profile trend. The results obtained from mineral analysis showed significant improvement in the calcium and phosphorus contents as major bone minerals of (T1) sesame seed supplement. Addition of nuts in prepared diet plan (T2) in the presence of 35% fenugreek amplified the omega fatty acids, linoleic, and linolenic contents that ultimately increased bone turnover potential. Amino acid profile of prepared product showed that substitution of nuts and used botanical seeds significantly improved the essential amino acid value that plays effective role in collagen structure during fracture healing. Oxidation inhibition activity of (T2) fenugreek‐based supplement observed higher reduce the oxidative stress and enhance the product shelf life. Overall, improved nutrition of products has positive effect on availability and absorption of each nutrient.

## CONFLICT OF INTERESTS

The authors declare no conflict of interest.

## ETHICAL APPROVAL

This study has nothing to do with human and animal testing.

## DECLARATION

The authors did not use the human subjects.
